# Rapid Target Binding and Cargo Release of Activatable Liposomes Bearing HER2 and FAP Single-Chain Antibody Fragments Reveal Potentials for Image-Guided Delivery to Tumors

**DOI:** 10.3390/pharmaceutics12100972

**Published:** 2020-10-15

**Authors:** Felista L. Tansi, Ronny Rüger, Claudia Böhm, Frank Steiniger, Martin Raasch, Alexander S. Mosig, Roland E. Kontermann, Ulf K. Teichgräber, Alfred Fahr, Ingrid Hilger

**Affiliations:** 1 Department of Experimental Radiology, Institute of Diagnostic and Interventional Radiology, Jena University Hospital, Friedrich Schiller University Jena, Am Klinikum 1, 07747 Jena, Germany; MusiClaudia@t-online.de (C.B.); ulf.teichgraeber@med.uni-jena.de (U.K.T.); 2 Department of Pharmaceutical Technology, Friedrich-Schiller-University Jena, Lessingstrasse 8, 07743 Jena, Germany; alfred.fahr@uni-jena.de; 3 Center for Electron Microscopy, Jena University Hospital, Friedrich Schiller University Jena, Ziegelmuehlenweg 1, 07743 Jena, Germany; frank.steiniger@med.uni-jena.de; 4 Center for Sepsis Control and Care, Institute of Biochemistry II, Jena University Hospital, Friedrich Schiller University Jena, Am Klinikum 1, 07747 Jena, Germany; martin.raasch@med.uni-jena.de (M.R.); Alexander.Mosig@med.uni-jena.de (A.S.M.); 5 Institute of Cell Biology and Immunology, University Stuttgart, Allmandring 31, 70569 Stuttgart, Germany; roland.kontermann@izi.uni-stuttgart.de

**Keywords:** liposomes, fluorescence quenching, optical imaging, molecular targeting, HER2, tumor microenvironment, tumor heterogeneity

## Abstract

Liposomes represent suitable tools for the diagnosis and treatment of a variety of diseases, including cancers. To study the role of the human epidermal growth factor receptor 2 (HER2) as target in cancer imaging and image-guided deliveries, liposomes were encapsulated with an intrinsically quenched concentration of a near-infrared fluorescent dye in their aqueous interior. This resulted in quenched liposomes (termed LipQ), that were fluorescent exclusively upon degradation, dye release, and activation. The liposomes carried an always-on green fluorescent phospholipid in the lipid layer to enable tracking of intact liposomes. Additionally, they were functionalized with single-chain antibody fragments directed to fibroblast activation protein (FAP), a marker of stromal fibroblasts of most epithelial cancers, and to HER2, whose overexpression in 20–30% of all breast cancers and many other cancer types is associated with a poor treatment outcome and relapse. We show that both monospecific (HER2-IL) and bispecific (Bi-FAP/HER2-IL) formulations are quenched and undergo HER2-dependent rapid uptake and cargo release in cultured target cells and tumor models in mice. Thereby, tumor fluorescence was retained in whole-body NIRF imaging for 32–48 h post-injection. Opposed to cell culture studies, Bi-FAP/HER2-IL-based live confocal microscopy of a high HER2-expressing tumor revealed nuclear delivery of the encapsulated dye. Thus, the liposomes have potentials for image-guided nuclear delivery of therapeutics, and also for intraoperative delineation of tumors, metastasis, and tumor margins.

## 1. Introduction

Optical fluorescence imaging in combination with drug delivery systems unifies properties that can advance image-guided medicine. These features can be exploited for example to promote early cancer diagnosis based on fluorescence imaging and to enhance the real-time visualization of tumor margins during oncologic surgery [1,2]. Due to their biocompatibility, liposomes which are lipidic nanovesicles that mimic biological membranes are widely used for the delivery of drugs or contrast agents, both in clinical and preclinical setups [3,4,5]. Their functionalization with targeting ligands drives the delivery of their cargo payload selectively to designated target cells, including tumor cells and components of the tumor microenvironment [6,7,8]. Thus, researchers around the globe have made relentless efforts to design liposomal formulations suitable for the targeting of conspicuous heterogeneous tumor markers. The human epidermal growth factor receptor 2 (HER2), for example, represents such a conspicuous heterogeneous tumor marker that has attracted research interest.

HER2 is a member of the epidermal growth factor receptor (EGFR) family of tyrosin kinases, and serves as a coreceptor for a variety of stromal ligands [9,10]. Its overexpression on 20–30% of breast cancers [11,12], esophageal, gastric [13], ovarian [14], salivary gland [15], and prostate [16] cancers, has been linked with an overall inadequate response to therapy, increased cancer relapse, and high mortalities and morbidities [17]. Targeted adjuvant therapies with HER2 humanized monoclonal antibodies such as trastuzumab, in combination with chemotherapy have improved the outcome in many patients with HER2 overexpressing tumors [18]. Nevertheless, a prominent trastuzumab resistance occurs in many patients, due to the inability of the antibody to target nuclear-localized HER2 protein [12]. This exposes that new strategies are still needed to target primarily the nuclear HER2 and reduce the high mortalities seen in HER2 overexpressing tumors especially breast carcinomas [19].

A known peculiarity of HER2 targeting antibodies is rapid binding. This was demonstrated with whole antibodies and also fragments thereof, either used as free molecules or conjugated to liposomal vesicles [4,20,21,22]. Interestingly, a rapid internalization of the antibodies and fragments thereof occurs following their binding to HER2 on the cell membrane, and correlates well with the internalization of the HER2 receptor itself [23,24,25]. Reports also indicate that HER2 is recycled back to the cell membrane after ligand-mediated internalization or degraded within the cells [4]. The kinetics of both processes can influence the binding and internalization of further ligands to the cells. This could partly be why the high affinity of radiolabeled HER2 single-chain antibody fragments (scFv) showed limited penetration into tumors [21]. Interestingly, liposomes targeted with whole HER2 antibodies or antibody fragments reveal increased nuclear localization of doxorubicin and depletion of tumor cells [4,5]. Considering that free doxorubicin internalized into cells eventually localize in the tumors, it is not clear if HER2 targeted liposomes would generally target substances into the nuclei of cells. Our previous work showed that trastuzumab-based liposomes deliver dyes into the nuclei of target tumor cells more efficiently in the in vivo situation, and to a lesser degree in vitro, whereas HER2-scFv based liposomes did not internalize cargos into the nuclei in cultured cells [22] and hence were excluded from in vivo studies then.

In the underlying study, we validated the ability of HER2’scFv-targeted liposomes to deliver fluorescent cargos into target cancer cells and enhance fluorescence imaging of xenograft models in mice. Another critical emphasis was to know whether bispecific targeting of liposomes with HER2’scFv together with FAP specific scFv would influence HER2’scFv based binding, and if the liposomes would deliver cargos into the nuclei of tumor cells in vivo similar to trastuzumab-targeting liposomes. The fibroblast activation protein (FAP) is a transmembrane sialoglycoprotein member of the dipeptidyl peptidase (DPIV) family and opposed to its other family members conveys collagenase and gelatinase activity [26]. Its expression is restricted to pathological conditions, such as on activated fibroblasts in healing wounds [27], rheumatoid arthritis [28], and on 90% of tumor-associated fibroblasts (TAFs) of many epithelial tumors [29]. In tumors, overexpression of FAP by TAFs is associated with excessive tumor growth and formation of metastasis [30], which makes it an attractive target for diagnosis and therapy [31]. Interestingly, human and murine FAP have 89% amino acid sequence homology and antibody cross-reactivity as a consequence [32]. Hence, we envisioned that simultaneous targeting of liposomes with FAP’scFv and HER2’scFv will potentially impact the HER2’scFv based rapid binding and deliver fluorescent cargos into cancer cells in vivo and enhance their fluorescence detection.

To accomplish this, quenched PEGylated liposomes were prepared by the lipid film hydration method whereby the intrinsically quenched near-infrared fluorescent (NIRF) dye, DY676-COOH at high concentrations was used in the hydration solution. In previous reports, we showed that quenched liposomes (termed LipQ) are activatable upon degradation and dye release and hence can enable exclusive detection of cells that take them up [33]. To be able to track intact liposomes within target cells before their degradation and activation, a green fluorescent phospholipid was embedded in the lipid bilayer. Subsequently, the liposomes were post-inserted with HER2’scFv and FAP’scFv conjugated micelles. The resulting monospecific (termed HER2-IL and FAP-IL) and bispecific liposomes (termed Bi-FAP/HER2-IL) were subjected to physicochemical characterization, cell uptake validations, and in vivo fluorescence imaging of xenograft models in mice.

The results revealed retention of the fluorescene-quenching and activation property of all liposomes, irrespective of the targeting moiety, and indicated a HER2-dependent rapid uptake, processing, and cargo release from HER2-targeting monospecific and bispecific liposomes in cultured cells and also in xenograft models in mice. Whereas no prominent nuclear localization of the encapsulated dye was seen in vitro in cultured cells with all the liposomes, Bi-FAP/HER2-IL-based live confocal microscopy of the high HER2 expressing SK-BR3 xenograft model exposed nuclear delivery of the encapsulated dye in vivo. Furthermore, whole body NIRF imaging revealed retention of tumor fluorescence for 32–48 h post-injection in both low and high HER2 expressing as well as in FAP-expressing xenograft models. Thus, we are convinced that the liposomes bear potentials for image-guided delivery of therapeutics into the nuclei and can be used for the image-guided delineation of tumors, metastasis, and tumor margins during surgery.

## 2. Materials and Methods

### 2.1. Materials for Liposome Preparation

Phospholipids used were egg phosphatidylcholine (EPC), cholesterol (chol), 1,2-distearoyl-sn-glycero-3-phosphoethanolamine-*N*-[methoxy (polyethylene glycol)-2000] ammonium salt (mPEG2000-DSPE), and 1,2-dioleoyl-sn-glycero-3-phosphoethanolamine-*N*-(7-nitro-2-1,3-benzoxadiazol-4-yl) (ammonium salt) (NBD-DOPE). All were purchased from Lipoid GmbH (Ludwigshafen, Germany) and Avanti Polar Lipids (Alabaster, AL, USA). The detergent Tris (hydroxymethyl)-aminomethane (Tris) and 4-(1,1,3,3-Tetramethylbutyl)phenyl-polyethylene glycol (Triton-X100) was acquired from Sigma (Taufkirchen, Germany), whereas the near-infrared fluorescent (NIRF) dye, DY676-COOH was from DYOMICs GmbH (Jena, Germany).

### 2.2. Preparation and Physicochemical Characterization of Quenched Liposomes

Quenched liposomes (termed LipQ) were prepared by hydrating a lipid film (composition EPC:Chol:mPEG_2000_-DSPE at a molar ratio of 6.5:3:0.5 and 0.3 mol% of the lipophilic marker NBD-DOPE) with a high concentration (4–5 mM in 10 mM Tris pH 7.4) of the NIRF dye DY676-COOH (excitation/emission: 674 nm/699 nm) and subsequent extrusion to 120–140 nm vesicles as reported in detail previously [33]. The extruded vesicles were purified by gel-filtration, and characterized according to previous reports [6,33,34]. Their encapsulation within the aqueous interior of the liposomes renders the DY676-COOH intrinsically fluorescence-quenched, whereas the “always-on” green fluorescent phospholipid NBD-DOPE (excitation/emission: 480 nm/530 nm) embedded in the lipid bilayer enables tracing of intact liposomes in cells before their degradation, and activation of the DY676-COOH.

### 2.3. Functionalization of Quenched Liposomes with FAP and HER2-Single-Chain Antibody Fragments

Human FAP- and HER2-specific single-chain antibody fragments (scFv) were purified from the periplasma of *Escherichia coli* (*E. coli*) cultures by metal affinity chromatography as described in detail elsewhere [35] and covalently conjugated to MalPEG_2000_-DSPE micelles by maleimide coupling for 60 min at room temperature. Sodium dodecyl sulphate polyacrylamide gel electrophoresis was applied to validate the purity and coupling efficiency of the scFv and scFv-micelles, which were subsequently post-inserted into the preformed quenched liposomes (at 0.1 and 0.3 mol% MalPEG_2000_-scFv), respectively, at 50 °C for 60 min as reported earlier [6]. To get bispecific liposomes, both the HER2- and FAP scFv based micelles were post-inserted simultaneously as demonstrated earlier [22] to achieve a FAP’scFv and HER2’scFv ratio of 2:3 after considering the ligand concentration used and the conjugation and post-insertion efficiencies. A detailed estimation of the efficiency of the post-insertion process was shown earlier using micellar lipid titration protocols [36,37]. The resulting activatable (quenched) liposomes were termed FAP-IL, HER2-IL, and Bi-FAP/HER2-IL (abbreviated Bis-IL in the figures), respectively. FAP proteins of human and murine origin have a very high amino acid sequence homology. Consequently, the human FAP antibody reacts with murine FAP protein. This enables the use of human FAP’scFv to target murine FAP positive tumor stromal cells in xenograft models in mice. Human and murine HER2 proteins have negligible amino acid sequence homology and no antibody cross reactivity making the human HER2’scFv based liposomes selective for the human HER2.

To estimate the dye content, lipid concentration, size, zeta potential, and morphology of the liposomal vesicles all the liposomes were subjected to dynamic light scattering, spectrometry, and electron microscopy as reported in detail elsewhere [6].

### 2.4. Validation of the Fluorescence Quenching, Activation, and Stability of Liposomes

A blue-shift in the absorption wavelength and low fluorescence emission are properties of fluorescence-quenched probes, whereas a re-shift towards red wavelengths and increased fluorescence denote activation. Intact (storage at 4 °C) and freeze-damaged liposomes (freezing at −80 °C) were therefore subjected to absorption (Ultrospec 4000 photometer, Pharmacia Biotech, Markham, Ontario, Canada) and fluorescence emission (Jasco FP6200 spectrofluorometer, Jasco, Gross-Umstadt, Germany) measurements of the liposomes (at 200 nmol (final lipids) in 100 µL 10 mM Tris pH 7.4) to demonstrate this phenomenon according to previous reports [33]. To verify their stability, liposomes were treated with fetal calf serum prior to the measurements.

### 2.5. Cell Lines Used and Culture Conditions

The human breast carcinoma cell lines MCF-7, BT-474, and SKBR3 and the human melanoma cell line, MDA-MB435S were acquired from the Cell Lines Service, (CLS, Heidelberg, Germany), under the item numbers 300273, 300131, 300333 and 300277 respectively) and grown in Dulbecco’s modified Eagle’s medium, whereas the human FAP-transfected fibrosarcoma cell line, HT1080-hFAP was cultured in RPMI medium. All culture media were supplemented with 10% (*v*/*v*) fetal calf serum (Life Technologies GmbH, Darmstadt, Germany), and the cells grown under standard conditions (37 °C in a 5% CO_2_ and 95% humidity).

### 2.6. Liposomal Uptake in Cultured Target Cells under Static Conditions

The selectivity of liposomes to target expressing cells was verified under standard (static) culture conditions by qualitative (microscopic) and semiquantitative (macroscopic NIRF) imaging. For microscopy, 60,000 cells (SKBR3) or 30,000 cells (HT1080-hFAP, MDA-MB435S, and MCF-7) were seeded on poly-l-lysine-coated 8-well culture slides (BD Biosciences Europe, Erembodegem, Belgium) and grown for 16 h then subsequently exposed to 200 nmol (final lipid) of the respective activatable liposomes for different durations at 37 °C. Subsequent steps for cell fixation, nuclear stain, and confocal microscopy were done on an LSM510 (Zeiss, Jena, Germany) as reported earlier [6]. For macroscopic NIRF imaging, 2 × 10^6^ cells (SK-BR3, MCF-7, HT1080-FAP, and MDA-MB435S) were seeded and grown overnight in small tissue culture flasks under standard conditions, then exposed to 200 nmol (final lipids) of the respective liposomes for different durations under standard culture conditions. Cells were harvested by washing two times with Hanks buffered salt solution (HBSS), scraping in HBSS, and pelleting by centrifugation at 200 g. The cell pellets were NIRF imaged with the red filterset in the Maestro™ fluorescence imaging system (CRi-InTAS, Woburn, MA, USA) according to previous reports [6].

### 2.7. Liposomal Uptake in HER2-Expressing Target Cells under Dynamic Flow Conditions

To verify the binding and cargo release property of HER2-targeting liposomes under flow conditions (HER2-IL and a control Trastuzumab-liposome, Tras-IL) the BT-474 breast cancer cell line which expresses a high level of HER2 and adheres on culture dishes better than the SK-BR3 was used. A total of 10,000 cells were seeded per well on a microfluidic chip (ChipShop, Jena, Germany) and grown overnight under static growth conditions. After that, the microfluidic chip was connected to the peristaltic pumps (Ismatec REGLO digital, Cole-Parmer, Wertheim, Germany) and the HER2-IL or the Tras-IL at 10 nmol/mL dissolved in cell culture media perfused for 15, 30, 45, and 60 min at three different shear stress rates of 0.7 dyn/cm^2^ typically observed for example in liver sinusoids, 3 dyn/cm^2^ found in the human suprarenal aorta [38], and 10 dyn/cm^2^ resembling conditions in the human common carotid artery [39]. Cells were subsequently fixed and subjected to fluorescence microscopy for detection of liposomal incorporated dyes as described previously [34]. Fluorescence intensities of the cells were deduced semiquantitatively with the ImageJ 1.46r (NIH, Bethesda, MD, USA) choosing five or more regions of interest.

### 2.8. Animal Studies

Studies on animals were approved by the regional animal committee (Thueringer Landesamt fuer Lebensmittelsicherheit und Verbraucherschutz, Bad Langensalza, Germany, project identification code 02-047/11, 1 December 2011), and conformed with international guidelines on the ethical use of animals. Female immune-deficient athymic nude mice (Hsd:Athymic Nude-Foxn1^nu^ nu/nu; Harlan Laboratories, Venray, The Netherlands) used were 8–12 weeks old during tumor induction (subcutaneous injection of 2.0 × 10^6^ cells in 120 µL cold Matrigel™ (BD Biosciences) 2–4 weeks prior to in vivo imaging) and 12–18 weeks old during imaging. Their body weights ranged from 22 to 24 g. Housing was done under standard conditions with mouse chow and water ad libitum.

### 2.9. Determination of Tumor Volumes

Tumor length, width, and height were acquired with a digital caliper and used to deduce the tumor volumes according to Feldmann [40].

### 2.10. Whole Body NIRF Imaging

Female nude mice bearing subcutaneous xenografts with diameters of the range 5–10 mm were subjected to tumor imaging under 2% isoflurane anesthesia. Thereby, 20 µmol/kg body weight (final lipids) of the respective liposomes in PBS were applied intravenously through the tail vein. The mice were imaged immediately after injection (time point, *t* = 0 h post-injection (p.i.)) and then every 2 h for 10 h and again at *t* = 24–32 h and at 48 h p.i. Images were acquired with the red filter set (Excitation range 615–665 nm and emission >700 nm (cut-in filter)) on the Maestro™ in vivo fluorescence imaging system (CRi-InTAS, Woburn, MA., USA) and evaluated by unmixing background auto-fluorescence acquired before probe injection on the Maestro 3.0 software (Version 2, CRi-InTAS, Woburn, MA., USA). The semiquantitative fluorescence intensities of regions of interest (ROIs) of tumors as compared to background (muscle; thigh region) were determined on each of the unmixed, intensity scaled (for exposure time, camera gain, binning and bit depth) tumors or muscle as reported earlier [6] and given as average signal (scaled counts/s) for comparison.

### 2.11. Monitoring Liposome Stability and Activation In Vivo by NIRF Imaging of Eyes

To monitor the serum stability of liposomes in vivo by imaging the eyes of mice as reported elsewhere [41], the respective liposomes (20 µmol final lipids/kg body weight) were applied intravenously in female nude mice and the eyes were imaged at designated time points on the Maestro™ in vivo fluorescence imaging system (CRi-InTAS, Woburn, MA, USA) using the red filterset as stated above. Further evaluation and determination of the fluorescence intensities of the eyes was done as similar to the whole body NIRF images stated above.

### 2.12. Euthanasia and Ex Vivo Biodistribution Studies

Mice were sacrificed with carbon dioxide after sedating with 2% isoflurane. The organs and tumors were collected and bio-optically imaged, then their fluorescence intensities semiquantitatively deduced as described above.

### 2.13. Statistical Evaluation

If not otherwise indicated Student’s *t*-test and 1-way ANOVA tests were used to estimate the level of significance, when normality and equal variance were applicable. Experiments were done at least three times. Animal studies included five or more mice per group. Differences deduced as *p* < 0.05 were considered significant.

## 3. Results

### 3.1. Preparation and Physicochemical Characterization of Activatable Liposomes

**Figure 1:**
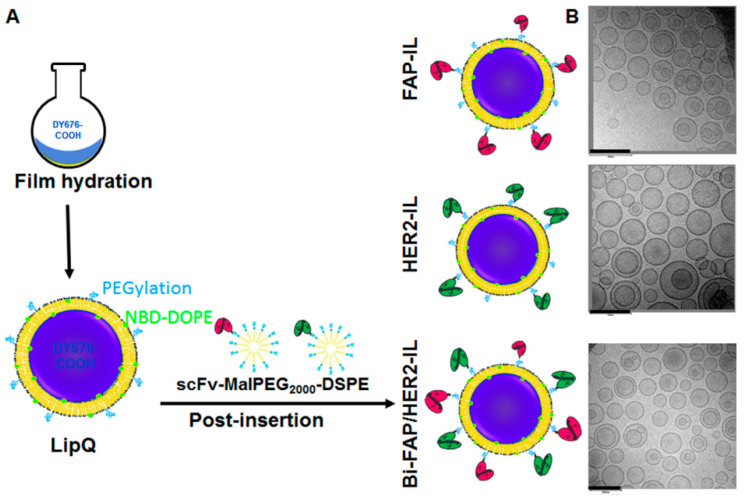
Scheme of the workflow for the liposomal preparation (**A**) and representative electron micrographs of the resulting quenched/activatable liposomes (**B**). Scale bar: 200 nm.

Fluorescence quenched liposomes (termed LipQ) were prepared by the film-hydration and extrusion method, whereby a lipid film was hydrated with a 4–5 mM concentration of the intrinsically quenched near-infrared fluorophore, DY676-COOH. In this setup, the liposomes carry the dye in the aqueous interior in its quenched state (Figure 1A, LipQ). Hence, the quenched dye grants the liposomes activatability upon triggering dye release, for example after degradation within cells, or by rupturing the liposomal lipid bilayer with organic solvents and other damaging conditions. After extrusion through a 100 nm polycarbonate membrane, the liposomes were post-inserted with micelles that were preconjugated to FAP- and/or HER2-specific scFv in order to make them selective for FAP and HER2 expressing cells. Monospecific liposomes were post-inserted with the respective FAP’scFv or HER2’scFv MALPEG_2000_-DSPE micelles (Figure 1A, FAP-IL and HER2-IL) whereas bispecific liposomes were post-inserted with both micelles (Figure 1A, Bi-FAP/HER2-IL). Analyses of the liposomal size, polydispersity indices, and zeta-potentials by dynamic light scattering revealed a relatively homogeneous distribution of vesicles in the range 120–140 nm diameters (Supplementary data S1), whereas electron microscopy substantiated the liposomal diameters of approximately 120 nm (Figure 1B).

### 3.2. The Quenched Liposomes Show Dye Activatability upon Damage and Relative Stability in Serum

**Figure 2:**
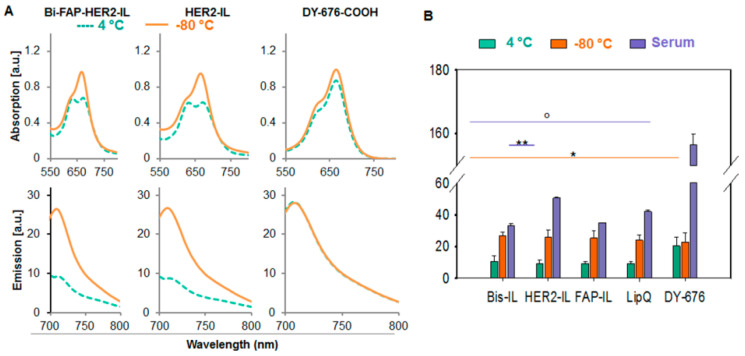
Validation of liposome quenching and activatability by spectroscopic measures. (**A**) The spectra of 200 nmol (final lipids) of the liposomes show double absorption maxima and low fluorescence emission when intact (green broken lines) and a single absorption maximum and increased fluorescence emission after freeze damage (orange lines). Contrarily, the free DY676-COOH measured at a concentration equivalent to the dye content in 200 nmol LipQ, shows only a single absorption peak and high fluorescence emission independent of the storage condition at 4 or −80 °C. (**B**) Semiquantitative levels of fluorescence emitted by intact (4 °C, green bars) and freeze-damaged liposomes (−80 °C, orange bars) reveal an almost 3-fold increase in liposomal fluorescence upon activation. Incubation with serum leads to a 3–4-fold increase in liposomal fluorescence (relative to the level of intact liposomes in buffer), and a 6-fold increase in fluorescence of the free DY676-COOH (relative to the level of the dye in buffer). Each bar depicts the mean of three independent (for 4 and −80 °C) or two measurements (serum) and the standard deviations. * *p* < 0.001 for freeze-damaged versus intact liposomes, ** *p* < 0.05 for HER2-IL in serum versus Bis-IL or FAP-IL in serum, ° *p* < 0.001 for DY676-COOH in serum versus all intact liposomes and liposomes in serum.

We verified the activation of the liposomal encapsulated dye upon triggering release after harsh freezing and thawing conditions. When diluted in buffer, intact liposomes showed double absorption maxima with blue-shifted wavelengths and relatively low fluorescence emission (Figure 2A, green broken lines), whereas freeze-damaged liposomes revealed a single absorption maximum and almost 3-fold increase in fluorescence emission resulting from dye release and activation (Figure 2A, orange lines). The lipid bilayers of liposomes get damaged and leaky by harsh freezing and thawing procedures. As a consequence, they release the encapsulated cargo into the surrounding solution after freeze-damaging, which results in fluorescence activation upon dilution of the released dye. Opposed to the liposomes, the free DY676-COOH at a concentration equivalent to the DY676-COOH content in 200 nmol (final lipids) of LipQ, showed a single absorption maximum and high fluorescence emission, irrespective of the freezing conditions. Thus, the liposomal formulations showed significantly lower (° *p* < 0.001) fluorescence intensities than the free DY676-COOH in their intact form (Figure 2B, green bars) and a significant increase in fluorescence intensity (* *p* < 0.001) after freeze-damaging (Figure 2B, orange bars) whereas the free DY676-COOH stored at 4 °C or frozen at −80 °C did not show any difference in fluorescence. Furthermore, incubation of the intact liposomes in 50% fetal calf serum for 24 h at 37 °C caused only mild increases in fluorescence emission as compared to the free DY676-COOH whose spectroscopic properties are greatly influenced by serum proteins. Consequently, the free dye revealed a 6-fold increase in fluorescence emission (relative to its fluorescence level in storage buffer) when diluted in serum (Figure 2B, purple bars). In contrary, the liposomes except for the HER2-IL which showed almost a 4-fold increase only revealed a 3-fold increase in fluorescence emission as compared to their intact states in buffer. Interestingly, the overall dye content in the liposomes would lead to a comparable level of fluorescence intensity like the free dye if released. This could be demonstrated in previous studies, whereby the liposomes were first freeze damaged then diluted in serum and immediately measured [22]. Thus, the results affirm the relative stability of the liposomes in serum, and the activation of the liposomal encapsulated dye exclusively upon release from the liposomes. This highlights their suitability for in vitro and in vivo applications in which intact and fluorescence-activated liposomes can be traced and distinguished.

### 3.3. HER2-scFv Bearing Activatable Liposomes Show a More Rapid Cargo Release and Activation than FAP-IL and Non-Targeted Liposomes In Vivo

**Figure 3:**
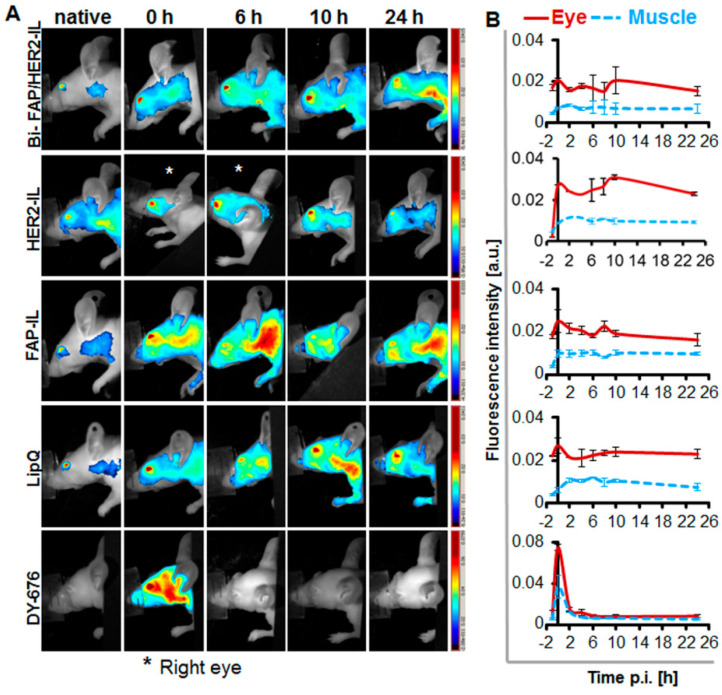
Fluorescence imaging of the eyes as a tool for monitoring the stability of fluorescence activatable liposomes in vivo during circulation. (**A**) NIRF images of the head region of the mice showing fluorescence of the eyes. (**B**) Semiquantitative levels of the fluorescence intensities of regions of interest (ROIs) placed in the eyes or background (back) of the mice. *n* = 2 ± SD.

Serum stability of liposomes was further verified in vivo by imaging the fluorescence emission of the eyes of nude mice lacking tumors, at designated time points post i.v. injection of probes as reported earlier [41]. The HER2-IL which revealed a comparably higher dye release in in vitro serum incubations than the Bi-FAP/HER2-IL and FAP-IL showed a slight increase in fluorescence intensities from 2 to 10 h post i.v. injection, suggesting its activation by some organs and a subsequent DY676-COOH release back into the circulation (Figure 3A,B). Opposed to this, the bispecific Bi-FAP/HER2-IL, FAP-IL, and control LipQ revealed only an initial residual fluorescence at time 0 h post-injection, which decreased slightly at 2 h p.i. and remained constant, experiencing a slight increase again at 8–10 h post-injection. The results expose a relatively low level of opsonization immediately after injection. This is thanks to the 5% PEGylation of the liposomes, and their consequent long circulation in the system till 8–10 h post-injection, and/or a slower activation and DY676-COOH release as a result of the absence of target structures (Figure 3A,B). Opposed to the liposomal probes, the free DY676-COOH used at equivalent concentrations as the dye content of the liposomes injected, revealed an initial 4-fold higher fluorescence than all the liposomes. Furthermore, the fluorescence of the free dye almost completely disappeared from the circulation within 2 h post-injection, which confirms the fluorescence quenching and stability of the liposomes.

### 3.4. HER2-scFv Bearing Activatable Liposomes Show Rapid Cargo Release in Cultured Cells under Static Conditions

**Figure 4:**
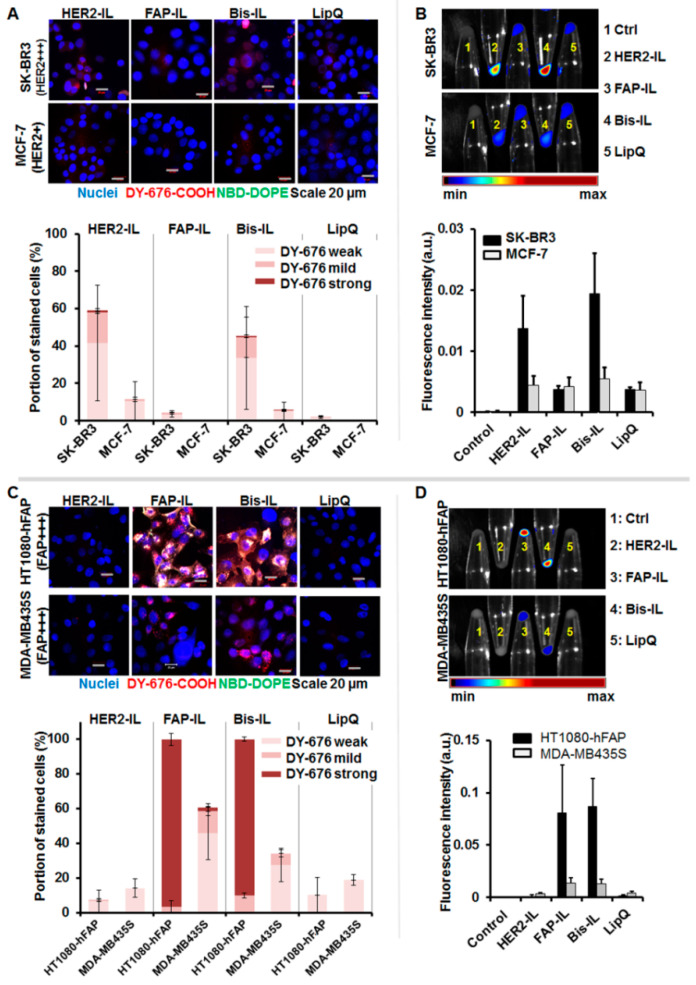
Confocal microscopy and NIRF analyses of liposomal cargo release in HER2 and FAP-expressing cells. Cells were incubated with 200 nmol of the indicated liposomes for 8 h at 37 °C. (**A**) Confocal microscopic images (scale bar 20 µm) and corresponding semiquantitation of the fluorescence intensities of the HER2 expressing cells reveal different levels of released/activated DY676-COOH in the cells (bars). This correlates with (**B**) the intensities of the DY676-COOH signals validated by macroscopic NIRF imaging of cell pellets. The Bi-FAP/HER2-IL (Bis-IL) and HER2-IL are taken up by SKBR3, whereas the FAP-IL and LipQ are not. (**C**) Confocal microscopic images (scale bar 20 µm) of FAP expressing cells reveal cells with different levels of released/activated DY676-COOH as determined by semiquantitation of the fluorescence intensities (bars). This correlated with the intensities of the DY676-COOH validated by macroscopic NIRF imaging of cell pellets (**D**). The Bi-FAP/HER2-IL (Bis-IL) and FAP-IL are selectively taken up by the cells, whereas the HER2-IL and LipQ are not taken up by the FAP-expressing cells. Each bar depicts the mean of three independent tests and the standard deviations.

The ability of the liposomes to be internalized and activated by target cells was validated qualitatively by confocal microscopy and semiquantitatively by near-infrared fluorescence imaging of cell pellets after incubation with the respective probes for 8 h at standard culture conditions. For this purpose, high HER2 expressing SK-BR3, low HER2 expressing MCF-7, high stable-FAP expressing fibrosarcoma HT1080-hFAP, and the low FAP expressing melanoma cell line MDA-MB435S were used. Incubation of the cells with the different liposomal formulations revealed varying levels of liposomal degradation and dye release. In accordance, only the HER2’scFv bearing liposomes (HER2-IL, Bi-FAP/HER2-IL) were taken up and activated by the HER2 expressing cells (Figure 4A,B) whereas the FAP-based formulations (FAP-IL, Bi-FAP/HER2-IL) were firmly taken up and activated by the HT1080-hFAP cells or mildly by the low FAP-expressing melanoma cell line, causing an intense red (DY676-COOH) and green (NBD-DOPE) fluorescence of the cells (Figure 4C,D). Interestingly, the HER2’scFv based (HER2-IL and Bi-FAP/HER2-IL) binding of HER2 on cells, and liposomal degradation and cargo release was relatively rapid, causing predominantly red fluorescence of the HER2-expressing cells in particular, and only minimal residual green fluorescence. The fact that the same bispecific Bi-FAP/HER2-IL bind FAP-specific cells stably in media containing serum confirms that the low fluorescence levels detected in the HER2-expressing cells is a consequence of rapid binding, dye release and recycling, and not serum related extracellular degradation of the liposomes. Furthermore, previous studies revealed the inability of the implemented cell lines to take up the free dye [22,34], and only negligible or no liposomal uptake was seen in the low HER2 (MCF-7) and low FAP (MDA-MB435S) expressing cell lines. Likewise, the non-targeted LipQ was not taken up by any of the cell lines (Figure 4A–D, LipQ).

### 3.5. HER2-scFv Bearing Activatable Liposomes Show Rapid Binding and Distinct Cargo Release under Dynamic Flow Conditions

Considering the rapid uptake and cargo release of the HER2-based liposomes (HER2-IL and Bi-FAP/HER2-IL) by high HER2 expressing cells under static conditions, we further investigated the uptake in dynamic cell culture experiments emulating conditions in the flowing blood. HER2-IL was compared with a liposomal formulation bearing the HER2-specific humanized therapeutic antibody, trastuzumab as target ligand. The liposomes were suspended in culture medium and perfused with defined shear stress rates (0.7, 3.0, and 10.0 dyn/cm^2^) over HER2 expressing BT-474 cells for 15, 30, 45, and 60 min respectively. Thereafter, the cells were fixed in the flow chambers and imaged on a fluorescence microscope.

**Figure 5:**
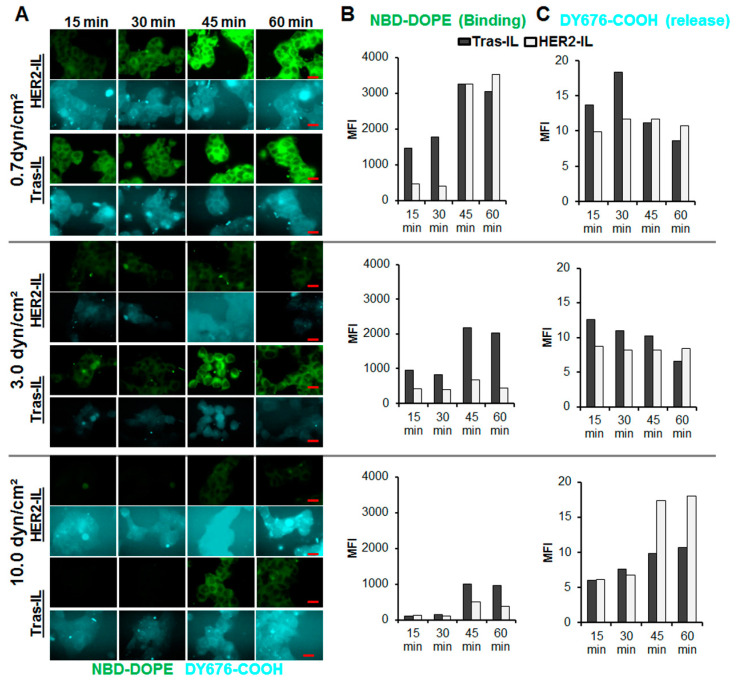
Validation of the interaction and cargo-release of HER2-targeting liposomes in HER2 expressing BT-474 cells under dynamic flow conditions. The HER2’scFv-bearing liposome, HER2-IL and a liposomal formulation bearing the HER2-specific humanized therapeutic antibody, trastuzumab (Tras-IL) were added to culture medium at 10 nmol (final lipids)/mL medium and flushed at the indicated flow rates (0.7, 3.0, and 10.0 dyn/cm^2^) over adherently growing BT-474 at standard culture conditions. At the indicated time points 15, 30, 45, and 60 min the perfusion was stopped, cells fixed in the flow chambers and imaged on a fluorescence microscope (**A**), scale bar, 20 µm. Thereafter, the images were subjected to semiquantitation of the green fluorescent phospholipid NBD-DOPE which is indicative of binding (**B**) and the liposomal encapsulated DY676-COOH which reveals liposomal cargo release and activation (**C**).

Cells treated with the liposomes revealed a rapid interaction, internalization, and subsequent release and activation of the encapsulated DY676-COOH. An intense green fluorescence of the cells was observed 15 min after incubation, in particular at a low shear stress rates of 0.7 dyn/cm^2^ (Figure 5A,B). Furthermore, the stability of binding of the probes to the cells, as determined by the level of the liposomal green fluorescent phospholipid (NBD-DOPE) in the cells, reduced with increase in flow rates (Figure 5B). This was more pronounced for the HER2-IL than for the Tras-IL. Interestingly, the cells also revealed fluorescence of the encapsulated DY676-COOH at the investigated time points (Figure 5A,C). The DY676-COOH was partially released into the surrounding medium in particular for HER2-IL compared to Tras-IL treatment. However, semiquantitation of the DY676-COOH fluorescence intensities within the cells over time revealed that the flow rate had a lesser influence on the activation of liposomes after their cellular uptake and cargo release. Taken together, the results substantiate the suggestion that binding, processing, and cargo release of HER2’scFv bearing liposomes is very rapid especially in high HER2-expressing target cells.

### 3.6. The HER2’scFv Bearing Activatable Liposomes Enhance Fluorescence Imaging upon Cargo Release in HER2-Expressing Tumor Models in Mice

**Figure 6:**
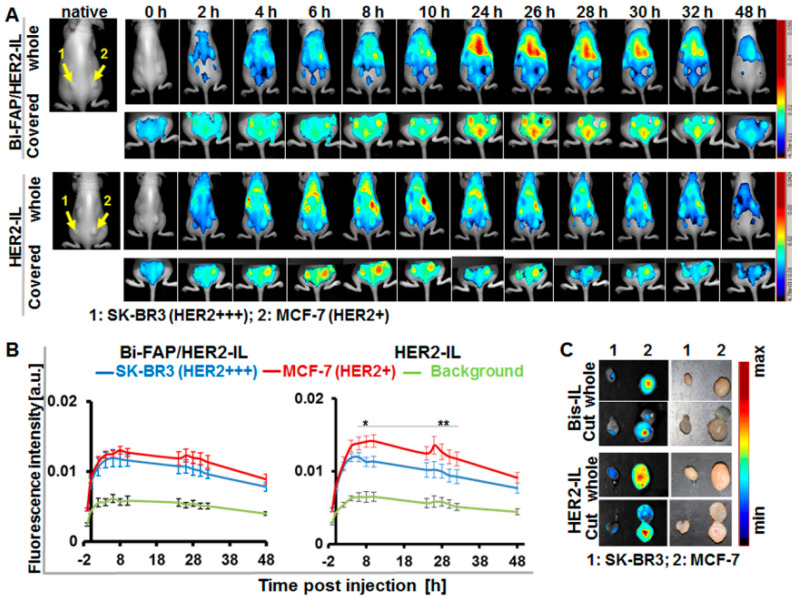
HER2’scFv-liposomes dependent cargo-release enhances NIRF detection of xenografted breast tumor models in mice. (**A**) Representative in vivo NIR-fluorescence images of mice bearing the high HER2-expressing human breast carcinoma model SK-BR3 (left) and low HER2 expressing MCF-7 (right) acquired with the red filter (Exc. 615–665 nm, Em cut-in, >700 nm) at 0–48 h post-injection of the indicated liposomes (Bi-FAP/HER2-IL and the HER2-IL). Images acquired after covering the kidneys and stomach clearly reveal the strong signals of the tumors, which peak at 24–26 h post-injection for Bi-FAP/HER2-IL (in both tumor models) and at 4–6 or 8–10 h for HER2-IL in the SK-BR3 and MCF-7 models, respectively. (**B**) Semiquantitative analysis of fluorescence intensities of tumors at the given time points post-injection. Each point depicts the mean of fluorescence intensities of regions of interest (ROIs) placed on the respective tumors (SK-BR3 and MCF-7) or background (thigh region) at the indicated time points after unmixing the auto-fluorescence. Each group constitutes *n* = 5 mice ± SEM. *p* < 0.05 for MCF-7 versus SK-BR3 at time points 8–10 (*) and 26–32 h (**) post-injection of HER2-IL, whereas accumulation of Bi-FAP/HER2-IL in SK-BR3 and MCF-7 models show no significant difference. (**C**) Representative NIR-fluorescence images and photographs of tumors (whole and cut) excised at 48 h post liposomal injection still reveal liposomal fluorescence.

Next we validated the ability of the HER2’scFv bearing activatable liposomes to accumulate in tumors, release their cargo (DY676-COOH), and subsequently enhance tumor imaging in vivo in mice models. Mice bearing both the high HER2-expressing SKBR3 and low HER2 expressing MCF-7 tumor models were injected with the respective liposomes (20 µmol (lipids)/kg weight) and imaged by whole body NIR-fluorescence imaging with the Maestro™ in vivo fluorescence imaging system. Injection of the bispecific Bi-FAP/HER2-IL revealed a gradual and continuous increase in the NIR fluorescence signals over time, in both the SK-BR3 and the MCF-7 tumors (Figure 6A). The tumor fluorescence signals were more visible after covering the kidneys and stomach of the mice (Figure 6A, covered) and peaked in both tumors between 24 and 26 h post-injection, as determined by semiquantitative analyses (Figure 6B). The gradual Bi-FAP/HER2-IL accumulation in the SK-BR3 and MCF-7 is based on both HER2 and FAP-directed binding of tumor cells and tumor associated fibroblasts. In contrast, the HER2-IL accumulated more in the low HER2 expressing MCF-7 than in the high HER2 expressing SK-BR3 (Figure 6A,B). The HER2-IL cargo release was quite rapid in both tumor models. Consequently, it revealed fluorescence signals that peaked at 4–6 h post-application for the SK-BR3 model and at 8–10 h post-injection for the MCF-7 model (Figure 6B). Interestingly, early accumulation in tumors shortly after injection is typical for probes which find targets directly on, or close to the tumor blood vessels or those with very rapid binding abilities. Hence, the slow cargo release of the Bi-FAP/HER2-IL as compared to HER2-IL is a result of bispecific targeting of both the HER2 and FAP within the tumors. Moreover, the liposomal NIR fluorescence signals was retained in tumors excised 48 h post-injection (Figure 6C), which indicates that the tumors will be detected in intraoperative setups even at this time point when the background fluorescence is low.

### 3.7. Role of HER2’scFv on Cargo Release of Bispecific HER2 and FAP Targeting Liposomes and Subsequent Imaging of High FAP Expressing Tumors

**Figure 7:**
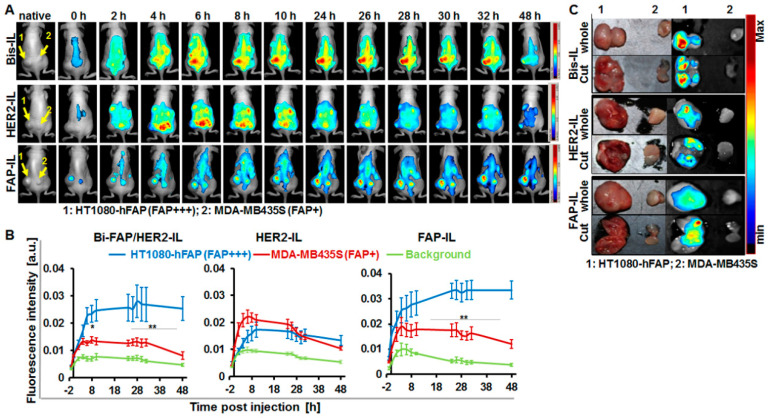
NIRF-imaging of FAP-expressing tumor models as a measure of cargo-release by FAP’ and HER2’scFv-bearing liposomes. (**A**) Representative in vivo NIR-fluorescence images of mice bearing stable human FAP-expressing fibrosarcoma HT1080-hFAP (FAP+++, left) and endogenous FAP-expressing human melanoma MDA-MB435S (FAP+, right) acquired with the red filter at 0–48 h post intravenous injection of the indicated probes (Bi-FAP/HER2-IL, HER2-IL, and FAP-IL). (**B**) Semiquantitative analysis of fluorescence intensities of tumors at designated time points post probe injection. Each point represents the mean of fluorescence intensities of regions of interest (ROIs) placed on the respective tumors (HT1080-hFAP and MDA-MB435S) or background (thigh region) at the indicated time points. *n* = 5 mice ± SEM. ** *p* < 0.05 for MDA-MB435S versus HT1080-hFAP at time points 2–8 h post-injection of HER2-IL. ** *p* < 0.05 for HT1080-hFAP versus MDA-MB435S at time points 24–48 h post-injection of FAP-IL. ** *p* < 0.05 for HT1080-hFAP versus MDA-MB435S at time points 8–48 h post-injection of Bi-FAP/HER2-IL. (**C**) Representative NIR-fluorescence images and photographs of tumors excised at 48 h post liposomal injection still reveal intense liposomal fluorescence of the high stable FAP expressing HT1080-hFAP.

We further validated the distribution of the HER2’scFv based liposomes in FAP expressing tumors. Animals bearing the stable FAP expressing fibrosarcoma (HT1080-hFAP) and the low endogenous FAP expressing melanoma (MDA-MB435S) models were injected with the respective liposomes (20 µmol (lipids)/kg weight) and imaged with the Maestro™ in vivo fluorescence imaging system as stated above. The bispecific Bi-FAP/HER2-IL accumulation and cargo release in both tumor models was gradual and continuous, and caused higher fluorescence signals in the HT1080-hFAP tumor models than the melanoma model. In contrast, the monospecific HER2-IL accumulated and released the dye very rapidly and to a greater extent in the low FAP expressing MDA-MB435S tumors during the first 24 h (Figure 7A). Thus, the Bi-FAP/HER2-IL accumulation and cargo release was based predominantly on FAP-directed binding, and enhanced a maximum tumor fluorescence signal at 26 h post-injection (Figure 7A,B). Contrarily, the HER2-IL accumulation and cargo release is probably related to phagocytic uptake by macrophages (Figure 7A,B, HER2-IL). The rapid cargo release is a consequence of rapid degradation of the liposomes in the cells, and accounts for the high fluorescence increase in tumors following liposome injection. This is seen in the fact that the monospecific FAP-IL accumulated and was activated much slower in both tumor models as compared to the Bi-FAP/HER2-IL, and revealed continuous fluorescence increases of HT1080-hFAP up to 48 h post-injection (Figure 7A,B, FAP-IL). Interestingly, the tumors excised 48 h post liposome injection revealed fluorescence signals which were still very high in the HT1080-hFAP model and negligible in the MDA-MB435S, irrespective of the probe injected (Figure 7C). This affirms again that the liposomes are suitable for implementation in intraoperative setups for the fluorescence delineation of tumors and tumorous tissues from healthy tissues.

### 3.8. Bispecific Liposomes Bearing HER2 and FAP Specific scFv Undergo Hepatobiliary Elimination

**Figure 8:**
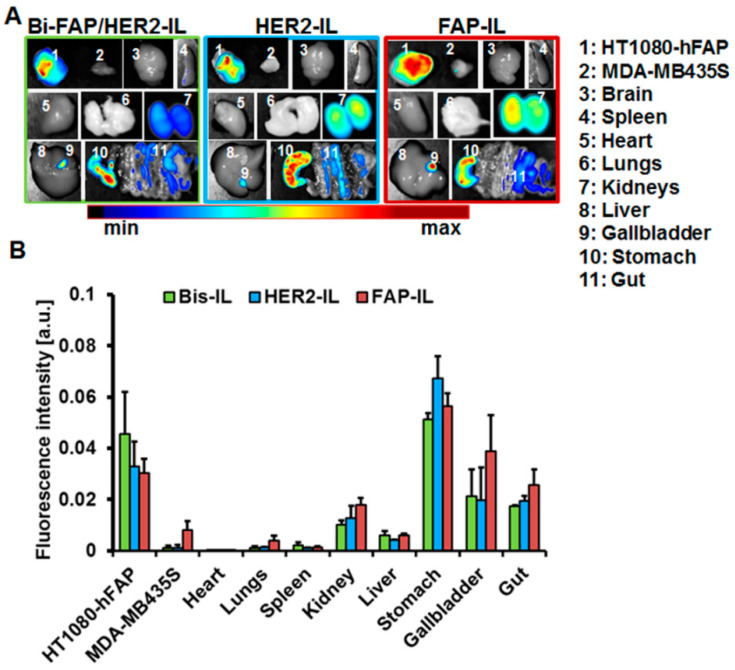
Biodistribution of HER2’ and FAP’scFv targeted liposomes in tumor bearing mice. (**A**) Representative NIR-fluorescence images of organs and tumors from mice bearing stable human FAP expressing fibrosarcoma HT1080-hFAP and endogenous FAP expressing human melanoma (MDA-MB435S) excised 48 h post intravenous injection of the indicated liposomes. (**B**) Semiquantitative analysis of fluorescence intensities of tumors at designated time points post probe injection. Each point represents the mean of fluorescence intensities of regions of interest (ROIs) placed on the respective tumors or organs. *n* = 5 mice ± SEM.

Mice organs were excised and imaged 48 h post-application of the indicated bispecific and monospecific liposomes and imaged. As can be seen in the NIR-fluorescence images of the organs (Figure 8A), fluorescence signals of the released liposomal DY676-COOH were evident mainly in the tumors and organs involved in liposomal elimination, such as the liver, gallbladder, kidneys, and the gastro-intestinal tract (gut). The fluorescence distribution pattern suggests both urinary and hepatobiliary elimination of the liposomal dye. At the investigated time point, the stable FAP expressing fibrosarcoma model is still strongly fluorescent as compared to the other organs, irrespective of the probe applied (Figure 8B). Interestingly, only the monospecific FAP-IL is detectable in the low endogenous FAP expressing melanoma model at this time. The influence of HER2’scFv fragments on the accumulation and cargo release of bispecific Bi-FAP/HER2-IL is again exposed in the level of fluorescence signals seen in tumors. The high tumor fluorescence at 48 h post-injection exposes a potential benefit of the liposomes for intraoperative imaging of tumors that can retain the liposomal dye for long durations, since the background fluorescence is minimal at this time.

## 4. Discussion

### 4.1. Physical Properties of the Liposomal Probes

Liposomes were prepared and encapsulated with a high concentration of a self-quenching NIRF dye, DY676-COOH as model for studying ligand-related cargo release. As shown previously, such quenched liposomes have potential for different biomedical applications including imaging and therapy monitoring as well as basic research on disease pathogenesis, primarily when targeted to different biomarkers overexpressed by tumor cells or the tumor stroma [7,22,33]. A striking peculiarity of the targeted liposomes is also the fact that they reveal individual characteristics of the targeting ligands used, which can range from speed of binding and ligand-related degradation and cargo-release in target cells. This is vital knowledge that is relevant to know when preparing liposomes for biomedical applications.

The underlying study demonstrates the influence of HER2 single-chain antibody fragments (HER2’scFv) on the uptake, degradation, cargo-release, and elimination of quenched liposomes, when used as a monospecific targeting ligand or in a bispecific targeting constellation together with FAP’scFv. Similar to previous reports, quenched liposomes encapsulated with a high concentration of the near-infrared fluorescent dye, DY676-COOH [33] could be easily furnished with the HER2’scFv alone or together with FAP’scFv by the post-insertion method. The liposomal sizes and polydispersity indices did not greatly change after the post-insertion process. All the targeted liposomes (bispecific Bi-FAP/HER2-IL and monospecific HER2-IL or FAP-IL) were similar to the non-targeted quenched liposomes (LipQ) which were used for the post-insertion based targeting and were in the range 120–140 nm as determined by dynamic light scattering and also transmission electron microscopy. Besides the size, the liposomes retained the quenched state and activatability of the encapsulated dye. Thus, liposomes stored at 4 °C, revealed fluorescence quenching in form of blue-shifted double absorption maxima and low fluorescence emission. Likewise, fluorescence activation following liposomal damage by harsh freezing procedures was seen in a red-shift of the absorption maximum and an almost 3-fold increase in fluorescence emission, a known characteristic of quenched fluorescent probes [42,43].

It is well accepted that a combination of π-stacking interaction and fluorescence resonance energy transfer (FRET) accounts for the self-quenching of the DY676-COOH. This occurs at high dye concentrations that bring the individual dye donor and acceptor species as close as molecule distances of below 100 Å [44]. As such the quenching is reversible upon dilution of the fluorophores to lower concentrations, or their interaction with protic substances or organic solvents [33], as could be shown for other fluorophores [42] and also upon dilution of the free dye with serum (Figure 2B). A significant advantage of fluorescent quenched liposomes is that they would permit fluorescence detection of the encapsulated dye exclusively upon release and activation, for example after uptake and degradation by target cells. Cells that take up the liposomes and those that process them and cause a fast cargo-release can be easily distinguished. Hence, fluorescence quenched and activatable HER2’scFv and FAP’scFv bearing bispecific Bi-FAP/HER2-IL could be exploited for tumor imaging purposes as demonstrated previously for other targeted quenched liposomes, and also for elucidating the features of the HER2’scFv as ligand for nano-therapeutics [34,43,45].

### 4.2. Selectivity of Bispecific Liposomes to Targets on Cultured Cells and Subsequent Cargo Release

Thanks to fluorescence quenching of the encapsulated NIRF DY676-COOH and also the presence of the green fluorescent phospholipid on the surface of the liposomes, their selectivity for target cells as well as their degradation, cargo-release, and activation of the encapsulated dye could be studied. At first glance, a rapid binding and processing of the HER2’scFv-based liposomes to target cells could be seen, which can be easily mistaken for instability. However, a strong liposomal green fluorescence of cells could be seen upon exposure to the HER2-IL under dynamic flow conditions. This indicated a rapid binding, as the green fluorescence increased over time from 15 to 60 min especially at low shear stress levels commonly found in liver sinusoids [38,39] (see Figure 5, 0.7 dyn/cm^2^). Considering that the cancer cells cannot take up the free dye components of the liposomes [22,34], the high DY676-COOH-based fluorescence seen in cells within 15 min exposure under flow conditions suggests a rapid binding, subsequent degradation and release of the encapsulated DY676-COOH within the cells (see Figure 5). In previous studies we showed that the liposomal components for example the green fluorescent phospholipids are recycled to the cell membrane and released from cells over time [33]. Thus, the intense green fluorescence of cells detected within 15 min under flow conditions and the predominant faint red fluorescence seen at later time points in target cells incubated under static conditions indicates that the HER2’scFv-based liposomes retain and combine rapid binding with a rapid internalization and cargo-release in HER2-expessing cells. This rapid processing is probably related to the HER2-receptor binding. Other researchers demonstrated similar rapid binding and internalization of liposomes based on whole antibodies or fragments thereof [4], and also revealed that HER2 protein is internalized upon ligand binding and can be recycled to the cell membrane or be degraded in the cells [24,25]. This strongly suggests that the HER2’scFv-based liposomes in the underlying work undergo HER2-dependent internalization and rapid cargo-release upon dissociation from the possibly cointernalized receptor. It is likely that some of the liposomal dye components are recycled out of the cells, as was evident in cell uptake under dynamic flow conditions. The fact that the Bi-FAP/HER2-IL are taken up stably by FAP-expressing cells further confirms that the observations in HER2-expressing cells is related to the HER2-target. Interestingly, the Bi-FAP/HER2-IL released their cargo more rapidly than the FAP-IL in FAP-expressing cancer cells, where they are taken-up based on the FAP expression levels (Supplementary data S3). Furthermore, the breast cancer cells and the endogeneous FAP-expressing melanoma (MDA-MB435S) show a higher retention of the encapsulated DY676-COOH than the green fluorescent phospholipid used to trace quenched liposomes, which further confirms a rapid binding, cargo release, and recycling of the liposomal components, especially the green phospholipid NBD-DOPE. Thus, the results further suggest that the different cell lines used have different levels of lipid metabolism and release from the cells, which could be vital for drug delivery when using liposomes.

### 4.3. Selectivity, Cargo Release, and Potential Applications of FAP/HER2 Targeting Liposomes In Vivo

In the in vivo situation using xenografted human cancer cells, various tumor components contribute to the accumulation, cargo release, and activation of quenched liposomes, alongside the target expression on the tumor cells themselves. These include the entire tumor microenvironment (TME, originating from the host mouse) and its constituents, such as the tumor vasculature which permits access of the liposomes to the tumors, the tumor associated macrophages (TAMs), which can phagocytose the liposomes irrespective of functionalization [34] and the tumor associated fibroblasts (TAFs) based on FAP expression. The level of TME cells are highly heterogeneous between tumor types, and greatly influence targeting of tumors with nanomedicines and imaging probes. Therefore, in the in vivo situation the bispecific Bi-FAP/HER2-IL should target the tumor cells (based on HER2 or human FAP), TAFs (based on murine FAP of host mouse) [6], and TAMs (based on phagocytosis) [33]. Depending on the tumor model, the diameter of nanomaterials for a suitable extravasation from the tumor vessels to the tumor cells and the TME is expected to be limited between 10 and 100 nm [46] or up to a maximum of 150 nm [7,22].

Similar to observations made with in vitro cell uptake studies, rapid binding and degradation and cargo-release of HER2’scFv based liposomes was retained in vivo. HER2-IL clearly detected both the high HER2 (SK-BR3) and low HER2-expressing (MCF-7) tumor models, as well as low FAP- (MDA-MB435S) and high FAP (HT1080-hFAP) expressing tumor models. In HER2 expressing tumors, the HER2-IL detected the MCF-7 tumors with low target abundance and more abundant blood vessels to a comparably higher level than the SK-BR3 model which has higher target abundance but lower vessel burden (see Supplementary data S2). This observation strongly suggests that, as would be expected for rapidly binding probes, the liposomes slowly gain access to more target cells in the higher vascularized tumors, since they distribute better within the tumor before being bound and degraded by target cells to invoke cargo-release and subsequent activation. Contrarily, tumor cells within the tumors with higher target abundance and low tumor vasculature like the SK-BR3 model are less accessible by highly affine probes, since the probes are trapped away immediately they extravasate the tumor blood vessels and encounter their targets [47]. Interestingly, reports show that high affinity restricted scFv molecules including a HER2’scFv-based radiolabeled probe from penetrating deeper into tumor tissues [21]. Nevertheless, based on our observations, the implementation of HER2’scFv-based liposomes could be beneficial for the rapid delivery of toxic therapeutics that aim to especially damage the tumor blood vessels. This would rely on the fact that the liposomal encapsulated content would be released in the immediate vicinity of the tumor blood vessels and could be retained there till they accomplish their effects on the cells. Interestingly, we previously reported affinity of trastuzumab targeted liposomes to perivascular components of the tumor vessels in mice models [22]. Whether this is the case with HER2’scFv-based liposomes is not entirely clear. The HER2-IL cargo release in higher vascularized, low FAP-expressing MDA-MB435S tumors was more rapid than in the lower vascularized, high FAP-expressing HT1080-hFAP models (see Figure 7). Both models have similar levels of tumor associated macrophages (not shown) and showed no expression of HER2 proteins in vitro. Furthermore, cross-reactivity of human HER2 targeting antibodies to the murine protein counterpart can be ruled out [48]. Thus, the higher vascular density and larger vessel lumens probably contribute to a faster access of TAMs coupled with a rapid cargo release of the HER2-IL within the TAMs in the melanoma model. This is evident in a rapidly increasing DY676-COOH based NIR fluorescence intensity till 8 h post-injection and subsequent decrease thereafter (see Figure 7B).

In contrast to the monospecific HER2-IL, the Bi-FAP/HER2-IL detected high or low HER2-expressing tumors to a similar level and high FAP expressing tumors dependent on the level of target expression. This suggests that bispecific targeting could be used in part as a strategy to influence the affinity of rapidly binding molecules. It is noteworthy that the HER2’scFv was inserted in the liposomes after site-directed maleimide coupling of a sulfhydryl group of a cysteine that was introduced to the C-terminus of the antibody fragment. Hence, a possible blockage of the binding moiety of HER2’scFv in both the monospecific and the bispecific liposomes can be excluded. Whether the slower accumulation of the bispecific probe in HER2-expressing tumors is due to a direct impact of the FAP’scFv antibody fragment on HER2’scFv or of the FAP’scFv on the HER2 receptor on target cells is not clear, and needs to be elucidated in the future.

Interestingly, live imaging of SKBR3 tumor revealed a deep penetration of Bi-FAP/HER2-IL shortly after injection, and also exposed the influence of the HER2’scFv on binding and cargo-release of the bispecific liposomes in vivo (Supplementary data S4, Video). Although breathing movements of the mouse partially distorted the image on the supplementary video, a rapid uptake of Bi-FAP/HER2-IL by tumor cells and pericytes located close to tumor vessels was evident in an intense green fluorescence of the liposomal phospholipid, NBD-DOPE. Furthermore, rapid release and activation of the liposomal DY676-COOH accounted for an intense red fluorescence of the tumor cells. This was particularly visible in tumor cells localized closest to large tumor blood vessels which the liposomes access first, and the intensity decreased from the blood vessels further towards less vascularized regions of the tumors. This strengthens the suggestion that although high ligand affinity could hinder the penetration of antibodies into tumors [21,47], the bispecific targeting of liposomes with HER2’scFv and other ligands such as the FAP’scFv used herein can modulate the affinity and permit deeper penetration of tumors, thereby retaining rapid cargo release based on specific ligand binding. Interestingly, the bispecific liposomes delivered the encapsulated DY676-COOH into the nuclei of the tumor cells similar to trastuzumab-based liposomes in previous reports [22], which indicates that the encapsulation of “nuclei active” therapeutic drugs within the bispecific liposomes also has potential to manage drug resistance in patients with high HER2 expressing tumors in the future. It could be shown that drug resistance, for example trastuzumab resistance in patients undergoing adjuvant treatment with trastuzumab and chemo- and other therapies results from the inaccessibility of nuclear localized targets [49]. Hence, we are convinced that the use of the Bi-FAP/HER2-IL conveys properties which can be combined for image-guided delivery of drugs to tumor and tumor microenvironment cells and also to manage resistance of drugs that exert their effects within the nuclei of tumor cells but are limited by restricted nuclear access. Furthermore, both the monospecific and bispecific liposomes convey potentials suitable for the image-guided delineation of tumors and tumorous margins during surgical interventions, as the liposomal dye is retained within tumors for up to 32–48 h post-injection (see Figure 6C and Figure 7C) whereas the released liposomal components are suitably eliminated via the hepatobiliary route.

## 5. Conclusions

Inserting HER2’scFv and FAP’scFv into activatable (quenched) liposomes led to influence of the binding properties of the ligands by each other. Bi-FAP/HER2 showed deep penetration into a poorly vascularized, high HER2-expressing tumor. Additionally, it retained a HER2-related rapid cargo release and subsequent activation which is beneficial for NIRF imaging and image-guided delivery. Furthermore, it delivered cargos into the nuclei of tumor cells in an in vivo breast tumor model. Taken together, the results highlight that the rapid binding monospecific HER2-IL would be suitable for image-guided drug delivery into low-HER2 expressing tumors, whereas the bispecific Bi-FAP/HER2-IL formulation can be modified for delivery into both high and low target expressing tumors. The liposomal drug delivery into the nuclei of tumor cells as seen in live imaging would abrogate resistance to nuclear active drugs which target the tumors but have limited access to the nucleus. Furthermore, both liposome formulations can enhance image-guided delineation of tumors and metastases in intraoperative procedures, and hence merit consideration in the future.

## Supplementary Materials

The following are available online at https://www.mdpi.com/1999-4923/12/10/972/s1, Table S1: Size increases of the liposomes were observed after post insertion of targeting antibodies, Figure S1: Relative levels of the vascular density of different xenograft models, Figure S2: Time course evaluations of liposomal uptake and cargo release in FAP expressing cells, Video S1: Material demonstrating the nuclear delivery of encapsulated DY676-COOH by Bi-FAP/HER2-IL.
