# Supplementary material for: Rapid Target Binding and Cargo Release of Activatable Liposomes Bearing HER2 and FAP Single-Chain Antibody Fragments Reveal Potentials for Image-Guided Delivery to Tumors
Source: Pharmaceutics. 2020 Oct 15;12(10):972. doi: 10.3390/pharmaceutics12100972 (PMC7650594; doi:10.3390/pharmaceutics12100972)
Supplement: Supplementary file 1 [file pharmaceutics-12-00972-s001.zip › Supplementary/pharmaceutics-960934-Supplementary.pdf]

Supplementary Materials: Rapid Target Binding and Cargo Release of Activatable Liposomes Bearing HER2 and FAP Single-Chain Antibody Fragments Reveal Potentials for Image-Guided Delivery to Tumors

Felista L. Tansi, Ronny Rüger, Claudia Böhm, Frank Steiniger, Martin Raasch, Alexander Mosig, Roland E. Kontermann, Ulf K. Teichgräber, Alfred Fahr and Ingrid Hilger

Supplementary data S1: Physicochemical characterization of liposomes by dynamic light scattering.

The sizes, polydispersity indices and zeta-potentials of liposomes were measured by dynamic light scattering. Size increases of the liposomes were observed after post insertion of targeting antibodies as shown in the Supplementary table 1 below.

Table S1. Size increases of the liposomes were observed after post insertion of targeting antibodies.

Parameter	Bi-FAP/HER2-IL	HER2-IL	FAP-IL	LipQ
**Size [nm]**	147.4 ± 4.8	138.7 ± 2.5	139.1 ± 2	129.6 ± 2
**PDI**	0.096 ± 0.1	0.064 ± 0.01	0.079 ± 0.02	0.05 ± 0.3
**Zeta Potential [mV]**	−15.1 ± 1.3	−14.6 ± 1.0	−15.4 ± 5	−18.6 ± 0.4

Supplementary data S2: Validation of the vascular densities of the tumor models

Chalkley estimation of the vessel marker CD31 was implemented to validate the level of vascularity as recommended by Vermeulen *et al* [1] and reported in detail elsewhere [2]. Microscopy of 3–7 specimens per xenograft model was performed, and 3 hotspots were analyzed per specimen. As seen in Error! Reference source not found., MCF-7 has a significantly higher vessel density (**P* = 0.0029) than SK-BR3, whereas the MDA-MB435 human melanoma revealed a significantly higher (***P* = 0.001) vascular burden than the HT1080-hFAP fibrosarcoma model. This partly explains the higher accumulation of HER2-IL in these tumor models than the high target positive models.

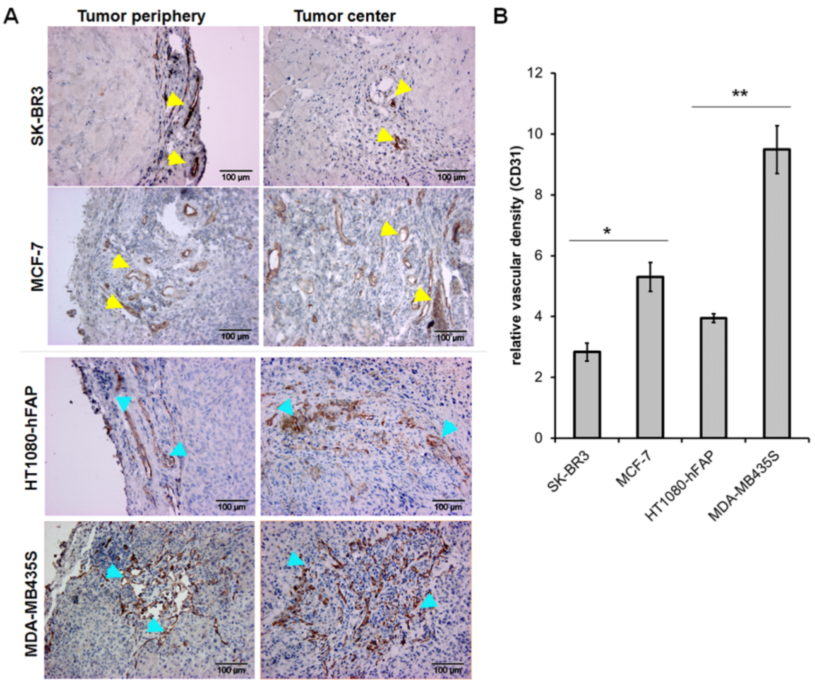

Figure S1. Relative levels of the vascular density of different xenograft models. **(**A) Representative pictures of CD31HOTSPOTS in human xenografted tumor models from mice. Arrowheads point at the respective vessels stained brown. Scale bar: 100 µm. **(**B) Relative levels of the CD31 in selected hotspots. Each bar denotes the mean counts from 3 hotspots of 6 (SKBR3 and MCF-7) or 4 (HT1080-hFAP and MDA-MB435S) independent specimens ± standard deviation. **P* = 0.0029 for MCF-7 versus SKBR3 whereas ** *P* = 0.001 for MDA-MB435S versus HT1080hFAP.

Supplementary data S3: HER2'scFv enhances rapid uptake and processing of Bi-FAP/HER2-IL by FAP expressing cells.

In FAP expressing target cells, the presence of HER2'scFv revealed a rapid uptake and processing of Bi-FAP/HER2-IL than the FAP-IL. The results suggest synergistic effects of the antibody fragments and confirm that the excessively rapid binding and processing in HER2-expressing tumor cells is related to HER2-ligand mediated internalization and recycling processes and not instability of the liposomes.

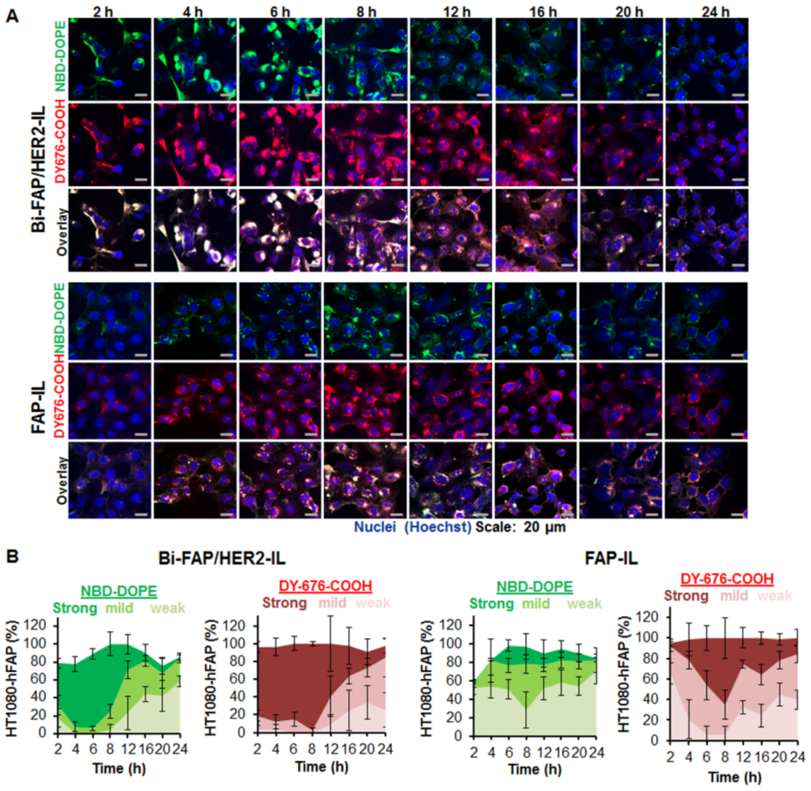

Figure S1: Time course evaluations of liposomal uptake and cargo release in FAP expressing cells. (A) Confocal microscopic images of the HT1080-hFAP cells cultured with BI-FAP/HER2-IL and FAP-IL for the indicated durations. (B) Semi-quantitative analysis of the relative abundance of the two liposomal dyes over the incubation duration. Whereas Bi-FAP/HER2-IL causes both strong NBD-DOE green fluorescence and DY676-COOH red fluorescence as from 2 h onwards, FAP-IL shows only mild green at red fluorescence at the beginning suggesting a more rapid uptake and processing due to HER2'scFv.

Supplementary data S4: Video material demonstrating the nuclear delivery of encapsulated DY676-COOH by Bi-FAP/HER2-IL.

Mice bearing the high HER2-expressing SK-BR3 breast cancer xenograft model was intravenously injected with 20 µmol /kg body weight (final lipids) of the Bi-FAP/HER2-IL, and the tumor imaged on an intravital microscope from approximately 10 to 60 min after injection. The short video demonstrates the rapid tumor penetration, binding and cargo release of the bispecific, Bi-FAP/HER2-IL. Deep penetration and uptake into the tumor cells and tumor fibroblasts is seen as intense green and increase in yellowish fluorescence, whereas rapid cargo release and activation is seen in the intense red fluorescence close to large accessory tumor blood vessels where initial liposomal access to tumors begin (visible at 25s and 50–58s of this video). The video shows distortions based on breathing movements of the mouse. This however, does not influence visualization of the probe fluorescence.

References

1. Vermeulen, P.B.; Gasparini, G.; Fox, S.B.; Colpaert, C.; Marson, L.P.; Gion, M.; Belien, J.A.; de Waal, R.M.; Van Marck, E.; Magnani, E., et al. Second international consensus on the methodology and criteria of evaluation of angiogenesis quantification in solid human tumours. *European journal of cancer* **2002**, *38*, 1564-1579.

2. Dhakal, H.P.; Naume, B.; Synnestvedt, M.; Borgen, E.; Kaaresen, R.; Schlichting, E.; Wiedswang, G.; Bassarova, A.; Giercksky, K.E.; Nesland, J.M. Vascularization in primary breast carcinomas: its prognostic significance and relationship with tumor cell dissemination. *Clinical cancer research : an official journal of the American Association for Cancer Research* **2008**, *14*, 2341-2350, doi:10.1158/1078-0432.CCR-07-4214.
