# Supplementary material for: Rapid Target Binding and Cargo Release of Activatable Liposomes Bearing HER2 and FAP Single-Chain Antibody Fragments Reveal Potentials for Image-Guided Delivery to Tumors
Source: Pharmaceutics. 2020 Oct 15;12(10):972. doi: 10.3390/pharmaceutics12100972 (PMC7650594; doi:10.3390/pharmaceutics12100972)
Supplement: Supplementary file 1 [file pharmaceutics-12-00972-s001.zip › Supplementary/Supplementary data S4-Video.pptx]

## Slide 1
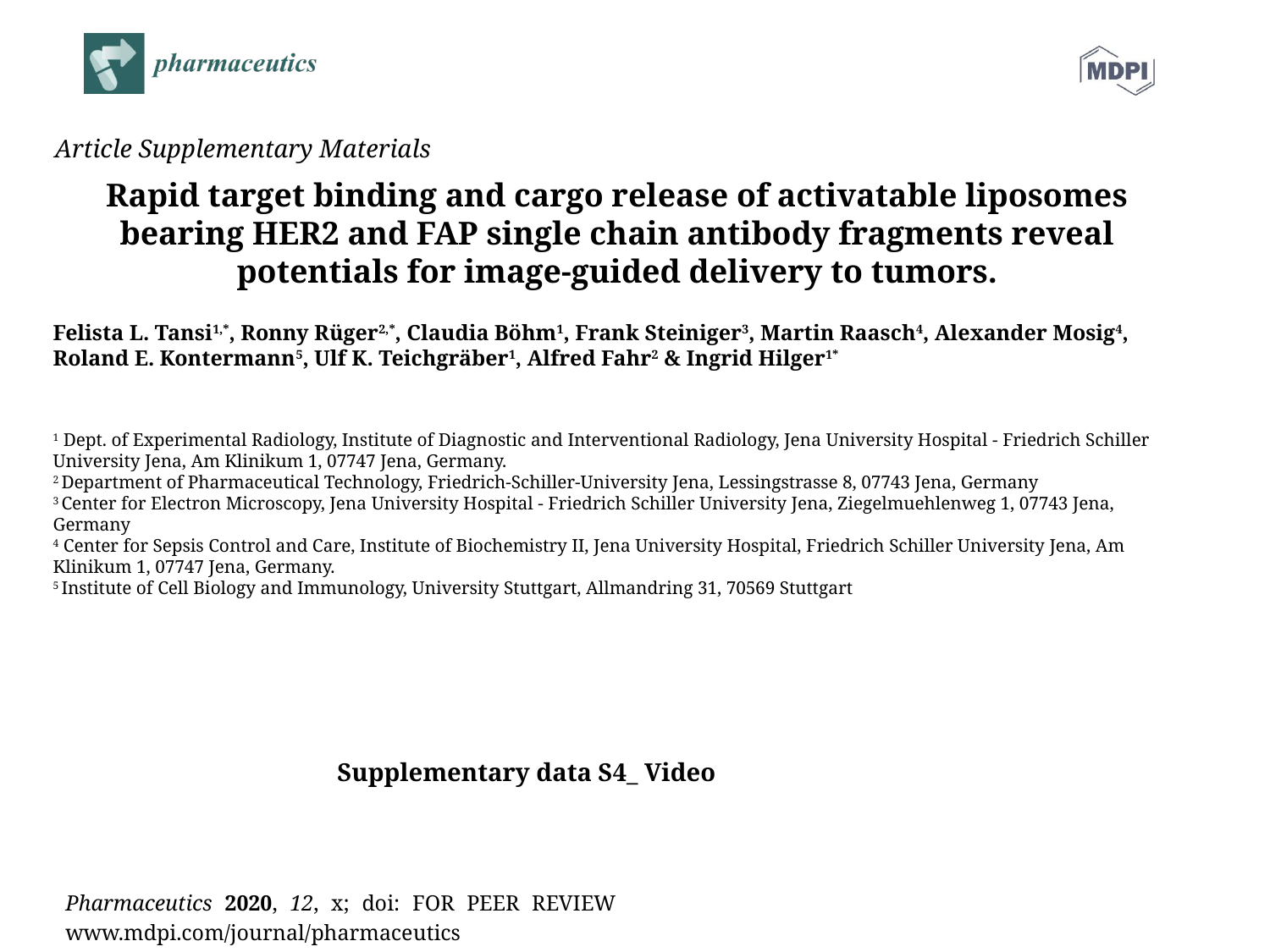

Article Supplementary Materials
Rapid target binding and cargo release of activatable liposomes bearing HER2 and FAP single chain antibody fragments reveal potentials for image-guided delivery to tumors.
Felista L. Tansi1,*, Ronny Rüger2,*, Claudia Böhm1, Frank Steiniger3, Martin Raasch4, Alexander Mosig4, Roland E. Kontermann5, Ulf K. Teichgräber1, Alfred Fahr2 & Ingrid Hilger1*
1 Dept. of Experimental Radiology, Institute of Diagnostic and Interventional Radiology, Jena University Hospital - Friedrich Schiller University Jena, Am Klinikum 1, 07747 Jena, Germany.
2 Department of Pharmaceutical Technology, Friedrich-Schiller-University Jena, Lessingstrasse 8, 07743 Jena, Germany
3 Center for Electron Microscopy, Jena University Hospital - Friedrich Schiller University Jena, Ziegelmuehlenweg 1, 07743 Jena, Germany
4 Center for Sepsis Control and Care, Institute of Biochemistry II, Jena University Hospital, Friedrich Schiller University Jena, Am Klinikum 1, 07747 Jena, Germany.
5 Institute of Cell Biology and Immunology, University Stuttgart, Allmandring 31, 70569 Stuttgart
Supplementary data S4_ Video
Pharmaceutics 2020, 12, x; doi: FOR PEER REVIEW www.mdpi.com/journal/pharmaceutics

## Slide 2
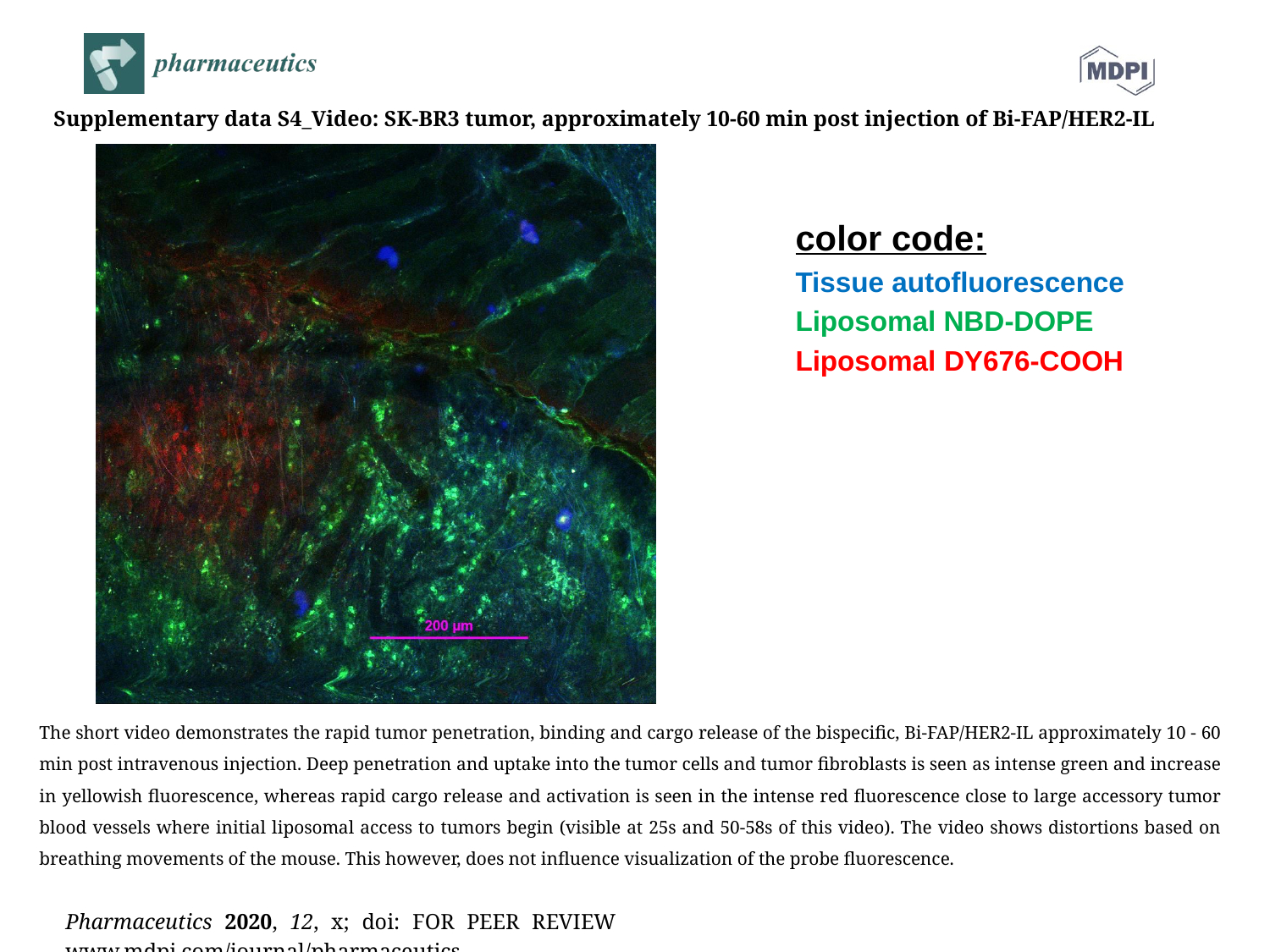

Supplementary data S4_Video: SK-BR3 tumor, approximately 10-60 min post injection of Bi-FAP/HER2-IL
color code:
Tissue autofluorescence
Liposomal NBD-DOPE
Liposomal DY676-COOH
The short video demonstrates the rapid tumor penetration, binding and cargo release of the bispecific, Bi-FAP/HER2-IL approximately 10 - 60 min post intravenous injection. Deep penetration and uptake into the tumor cells and tumor fibroblasts is seen as intense green and increase in yellowish fluorescence, whereas rapid cargo release and activation is seen in the intense red fluorescence close to large accessory tumor blood vessels where initial liposomal access to tumors begin (visible at 25s and 50-58s of this video). The video shows distortions based on breathing movements of the mouse. This however, does not influence visualization of the probe fluorescence.
Pharmaceutics 2020, 12, x; doi: FOR PEER REVIEW www.mdpi.com/journal/pharmaceutics
